# CircHIPK3/miR-876-5p/PIK3R1 axis regulates regulation proliferation, migration, invasion, and glutaminolysis in gastric cancer cells

**DOI:** 10.1186/s12935-020-01455-w

**Published:** 2020-08-13

**Authors:** Qingchun Li, Yuan Tian, Yun Liang, Chang Li

**Affiliations:** 1 Department of Gastrointestinal Colorectal and Anal Surgery, China-Japan Union Hospital of Jilin University, No. 126, Xiantai Street, Changchun, 130031 Jilin China; 2 Center of Physical Examination, China-Japan Union Hospital of Jilin University, Changchun, 130031 Jilin China

**Keywords:** CircRNA, ceRNA, Tumorigenic, Target

## Abstract

**Background:**

Circular RNAs (circRNAs) are a new group of non-coding RNAs that play vital roles in cancer occurrence, including gastric cancer (GC). Nevertheless, the role and underlying regulatory mechanisms of circHIPK3 in GC remain unclear.

**Methods:**

The expression levels of circHIPK3, miR-876-5p, and phosphoinositide-3-kinase regulatory subunit 1 (PIK3R1) were estimated by real-time quantitative polymerase chain reaction (RT-qPCR) assay. The proliferation, migration, and invasion of GC cells were determined by 3-(4, 5-dimethylthiazol-2-yl)-2, 5-diphenyl-2H-tetrazol-3-ium bromide (MTT) and transwell assay. Glutaminolysis of GC cells was assessed by measuring glutamine, glutamate, and α-ketoglutarate levels. The western blot was employed to examine the related-protein expression. The association between miR-876-5p and circHIPK3 or PIK3R1 was predicted and affirmed by bioinformatics database starBase v2.0 and dual-luciferase reporter assay, respectively. Eventually, the xenograft experiment was used to assess the role of circHIPK3 silencing in vivo.

**Results:**

CircHIPK3 was upregulated in GC tissues and cells compared with controls, and circHIPK3 was more resistance to RNase R than linear homeodomain interacting protein kinase 3 (HIPK3) mRNA. Silencing of circHIPK3 inhibited GC cells proliferation, migration, invasion, and glutaminolysis as well as tumor tumorigenic ability. Moreover, we also found that miR-876-5p, interacted with PIK3R1, was a target gene of circHIPK3. CircHIPK3 silencing induced effects on GC cells were abolished by silencing of miR-876-5p. In addition, upregulation of PIK3R1 inversed miR-876-5p overexpression-induced effects on GC cells.

**Conclusion:**

The circHIPK3 mediated the proliferation, migration, invasion, and glutaminolysis of GC cells partly through regulation of miR-876-5p/PIK3R1 axis by the mechanism of competing endogenous RNAs (ceRNA), indicating circHIPK3 was a GC-associated circRNA that promoted GC development.

## Highlights

1. CircHIPK3 is obviously overexpressed in gastric cancer tissues and cells.
2. Knockdown of circHIPK3 inhibits gastric cancer cells proliferation, migration, invasion, and glutaminolysis through miR-876-5p/PIK3R1 axis.
3. CircHIPK3 increases PIK3R1 expression by targeting miR-876-5p.

## Background

Gastric cancer (GC) is universal malignant tumor all over the world, ranking as the third leading reason of cancer-associated mortality [1]. According to statistics, there are 1,000,000 new cases and 783,000 mortalities of GC in 2018 [2]. The clinical outcomes of GC patients remain poor in most countries, although many advancements have been achieved in terms of technologic methods. Therefore, it is required to discover new diagnosis biomarkers and comprehend the pathophysiology of GC.

Circular RNAs (circRNAs) are a class of circularly configured RNA molecules, lacking 5′ to 3′ polar or polyadenylation tails [3]. Recent data showed that circRNAs were widely expressed in eukaryotes and could act as key regulators in multiple biological processes [4]. Coincidentally, numerous studies revealed that circRNAs were closely associated with the occurrence and progress of malignant tumors, including GC. For example, Rong et al. revealed that circPSMC3 was closely connected with the progression of GC by interacting miRNA-296-5p, indicating that circPSMC3 was novel a target for the therapy of GC [5]. In view of this, it is meaningful to excavate the relevant molecular mechanisms of circHIPK3 in GC. CircHIPK3 (hsa_circ_0000284) is derived from the homeodomain interacting protein kinase 3 (HIPK3) gene and located on chr11 (33307958–33309057). Evidence indicated that circHIPK3 facilitated colorectal cancer cells proliferation and metastasis [6]. In addition, circHIPK3 also was overexpressed in epithelial ovarian cancer, which was associated with poor prognosis of patients [7]. Nevertheless, it was uncertain whether circHIPK3 is associated with regulation of GC development.

Previously published studies have described that aberrant expression of miRNAs played vital role in tumorigenesis, drug-resistance, and immune response [8–10]. By complementary base pairing with the 3′untranslated region (UTR) of mRNA, miRNA triggered mRNA degradation or translational repression [11]. Furthermore, the tumor inhibition impacts of miR-876-5p were confirmed in many types of tumor cells, including lung cancer [12], hepatocellular carcinoma [13], and GC [14]. An extensive understanding of the function of miR-876-5p in GC was necessary.

Phosphoinositide-3-kinase (PI3K) regulatory subunit 1 (PIK3R1) was identified as a regulator of PI3K/protein kinase B (AKT) signal pathway that was important and complicated in tumorigenesis [15]. Furthermore, PIK3R1 was overexpressed in endometrial cancer cells, and upregulation of miR-495 impeded endometrial cancer cells proliferation while induced apoptosis by directly targeting PIK3R1. The further investigation of the molecular mechanisms of PIK3R1 in GC was required.

Currently, the study was aimed to explore the biological function and underlying mechanism of circHIPK3 in GC. We measured circHIPK3 expression in GC tissues samples and cells. Additionally, functional experiments were used to investigate the regulatory mechanisms of circHIPK3 in regulation proliferation, migration, invasion, and glutaminolysis in GC cells.

## Materials and methods

### Tissues collection

In total of 26 GC patients who had not received any preoperative treatments were registered in the present study. The GC tissues and contiguous noncancerous tissue samples were harvested from patients with surgery at China-Japan Union Hospital of Jilin University and then transferred to a − 80 °C refrigerator for further preservation until further use. All patients offered the written informed consents, and the research was permitted by the Ethics Committee of China-Japan Union Hospital of Jilin University.

### Cell culture

The human normal gastric epithelial cell line GES-1 and GC cells (HGC-27) were bought from China Life Science Academy (Shanghai, China). GC cells (AGS) were gained from the American Type Culture Collection (Rockville, MD, USA). Cells were cultivated in RPMI 1640 medium supplemented with penicillin (100 U/mL), streptomycin (100 mg/mL), and 10% fetal bovine serum (GIBCO BRL, Grand Island, NY, USA) in a suitable environment containing 5% CO_2_ at 37 °C.

### Real-time quantitative polymerase chain reaction (RT-qPCR)

RNA samples were isolated from GC tissues and cells using Trizol reagent (Takara, Dalian, China) in accordance with the manufacturer’s instructions. The complementary DNA was synthesized using 2 μg of RNA template as template and reverse transcription kit (Takara) or microRNA Reverse Transcription Kit (Qiagen, Hilden, Germany). All complementary DNA products were quantitatively analyzed using SYBR Green Real-Time PCR Master Mix (Qiagen) under ABI Prism 7900 real-time PCR system (Applied Biosystems, Foster City, CA, USA). The transcription levels of circHIPK3, HIPK3, miR-876-5p, and PIK3R1 were computed based on the 2^−ΔΔCt^ method. Notable, phosphate dehydrogenase (GAPDH) and small nuclear RNA U6 were used to as housekeeping genes.

The sequences of primers were listed:

circHIPK3 (F-5′-GGGTCGGCCAGTCATGTATC-3′; R-5′-ACACAACTGCTTGGCTCTACT-3′);

HIPK3 (F-5′-TCACAAGTCTTGGTCTACCCA-3′; R-5′-CACATAGGTCCGTGGATAGTTTC-3′);

miR-876-5p (F-5′-GCCGAGTGGATTTCTTTGTG-3′; R-5′-CTCAACTGGTGTCGTGGA-3′);

PIK3R1 (F-5′-ACCACTACCGGAATGAATCTCT-3′; R-5′-GGGATGTGCGGGTATATTCTTC-3′);

GAPDH (F-5′-TCCCATCACCATCTTCCAGG-3′; R-5′-GATGACCCTTTTGGCTCCC-3′);

U6 (F-5′-AACGCTTCACGAATTTGCGT-3′; R-5′-CTCGCTTCGGCAGCACA-3′).

### Transfection assay

Specific small interfering RNA (siRNA) against circHIPK3 (si-circHIPK3) and siRNA scrambled control (si-NC), and short hairpin RNA objecting circHIPK3 (sh-circHIPK3) and shRNA scrambled control (sh-NC) were purchased from GenePharma (Shanghai, China). The miR-876-5p mimic (miR-876-5p) and its negative control (miR-NC), and miR-876-5p inhibitor (anti-miR-876-5p) and its negative control (anti-miR-NC) were designed and acquired from Thermo Fisher Scientific (Waltham, MA, USA). HGC-27 and AGS cells were transfected with plasmids or oligonucleotides using Lipofecamine2000 (Invitrogen, Carlsbad, CA, USA). The sequences for si-circHIPK3, si-NC, sh-circHIPK3 and sh-NC were as follows: si-circHIPK3: 5′-CUACAGGUAUGGCCUCACA-3′, si-NC: 5′-UUCUCCGAACGUGUCACGUTT-3′, sh-circHIPK3: 5′-ccggCUACAGGUAUGGCCUCACAttcaagagaTGTGAGGCCATACCTGTAGTTTTTTGGTACC-3′, sh-NC: 5′- ccggUUCUCCGAACGUGUCACGUTTttcaagagaAATCGTGACACGTTCGGAGAATTTTTTGGTACC-3′. For overexpression of PIK3R1 (PIK3R1), the primers were used for amplification and then cloned in the mammalian expression pcDNA3.1 vector (Invitrogen), PIK3R1 F-5′-CCGGAATTCATGAGTGCTGAGGGGTACCAGTAC-3′; and R-5′-CCGCTCGAGATCGCCTCTGCTGTGCATATATA-3′.

### RNase R treatment

3 U/mg of RNase R (Epicenter, Madison, WI, USA) was applied to treat RNA for 15 min at 37 °C, using Mock as control.

### Cell proliferation assay

HGC-27 and AGS cells were seeded into the 96-well plates with a concentration of 3000 cells per well and incubated overnight. After transfection, 20 μL of 3-(4, 5-dimethylthiazol-2-yl)-2, 5-diphenyl-2H-tetrazol-3-ium bromide (MTT; Beyotime, Shanghai, China) was added into the 96-well plates at the designated time points. After further culturing for 4 h, the supernatants were replaced with 150 μL of dimethyl sulfoxide (DMSO). The cell viability was assessed by detecting optical density at wavelength of 490 under a microplate reader (Applied Biosystems).

### Transwell assay

The migrant and invasive capabilities of GC cells were measured using 24-well transwell chamber pro-adhered without Matrigel (Becton–Dickinson, San Jose, CA, USA) or with Matrigel. HGC-27 and AGS cells (5 × 10^4^) were re-suspended in 200 μL of free-serum medium and then added into the upper chamber. After culturing for 24 h, the migrated and invaded cells on the lower chamber were fixed with 4% formaldehyde and then stained by violet crystalline for 1 5 min. Subsequently, each well of transwell was observed and photographed using a microscope (Olympus, Tokyo, Japan).

### Analyses of glutamine, glutamate, and α-ketoglutarate (α-KG) levels

Transfected or non-transfected HGC-27 and AGS (1 × 10^4^/well) were seeded into 6-well plates and cultured for 24 h. Glutamine/Glutamate Determination Kit (Sigma-Aldrich, Merck KGaA, Darmstadt, Germany) was used for detection of concentrations of glutamine and glutamate based on user’s guideline. The relative level of α-KG level was assessed by the α-KG Assay Kit (Abcam, Cambridge, MA, USA) in compliance with the specification of manufacturer’s manuals.

### Western blot assay

Proteins were isolated from tissues and cell lysis from HGC-27 and AGS cells by Radio-Immunoprecipitation assay (RIPA) buffer (Beyotime). After that, 40 μg of total protein was segregated by 10% sodium dodecyl sulfate polyacrylamide gel electrophoresis, and then protein blots were transferred onto nitrocellulose membranes (Beyotime). After that, membranes were shaken in 5% skim milk solution, and then incubated with appropriate primary antibodies: anti-amino-acid transporter 2 (ASCT2; ab237704; 1:1000 dilution; Abcam), anti-glutaminase (GLS1; ab156876; 1:1000 dilution; Abcam), or anti-GAPDH (ab181602; 1:1000 dilution; Abcam). After washing, the membranes were reacted with secondary antibody (ab150077; 1:2000 dilution; Abcam). Protein bands were examined using the Western Blotting Detection Kit (Solarbio, Beijing, China) and the intensity of bands was quantified using Image Lab software 5.2 (Bio-Rad, Hercules, CA, USA).

### Dual-luciferase reporter assay

The miR-876-5p binding sites in circHIPK3 or 3′ UTR of PIK3R1 were predicted by bioinformatics databases starBase v2.0 (http://starbase.sysu.edu.cn/). The 3′UTR of PIK3R1 and circHIPK3 harboring either the miR-876-5p binding site or mutant were cloned into the pmirGLO luciferase vector (Promega, Madison, WI, USA), named as circHIPK3 WT, circHIPK3 MUT, PIK3R1 3′UTR WT, or PIK3R1 3′UTR MUT, respectively. HGC-27 and AGS cells were co-transfected with constructed vectors and miR-876-5p mimic or miR-NC. After incubation for 48 h. HGC-27 and AGS cells were lysed, and luciferase activity was checked using dual-luciferase reporter assay system (Promega).

### In vivo experiment

The lentiviral vectors with sh-circHIPK3 (sh-circHIPK3) or control (sh-NC) were constructed by GenePharma. A total of 12 male BALB/c nude mice were purchased from Shanghai Experimental Animal Center (4–5 weeks old; Shanghai, China) and then were divided into 2 groups (6 mice per group). Sh-circHIPK3 or sh-NC stably infected HGC-27 cells (5 × 10^6^ cells were suspended in 200 μL of FBS-free culture medium) were hypodermically inoculated left axillary region of male BALB/c nude mice. Tumor growth was measured every week based on V = 1/2 × abasedonb^2^ method [length (a) and width (b) length of the tumor]. At 35 days, subcutaneous tumors were removed for weight detection and further analysis. This study was directed with approval from the Institutional Animal Care and Use Committee of China-Japan Union Hospital of Jilin University.

### Statistical analysis

Statistical analyses were conducted by Student’s t-test and one-way analysis of variance using the SPSS 21.0 software (IBM, Somers, NY, USA). In addition, *P*-value less than 0.05 denoted significant. All data were exhibited as mean ± standard deviation. Pearson’s correlation assay was carried out to analyze the correlations relationship between miR-876-5p and circHIPK3 or PIK3R1 expression.

## Results

### CircHIPK3 was overexpressed in GC tissues and cells

**Fig. 1:**
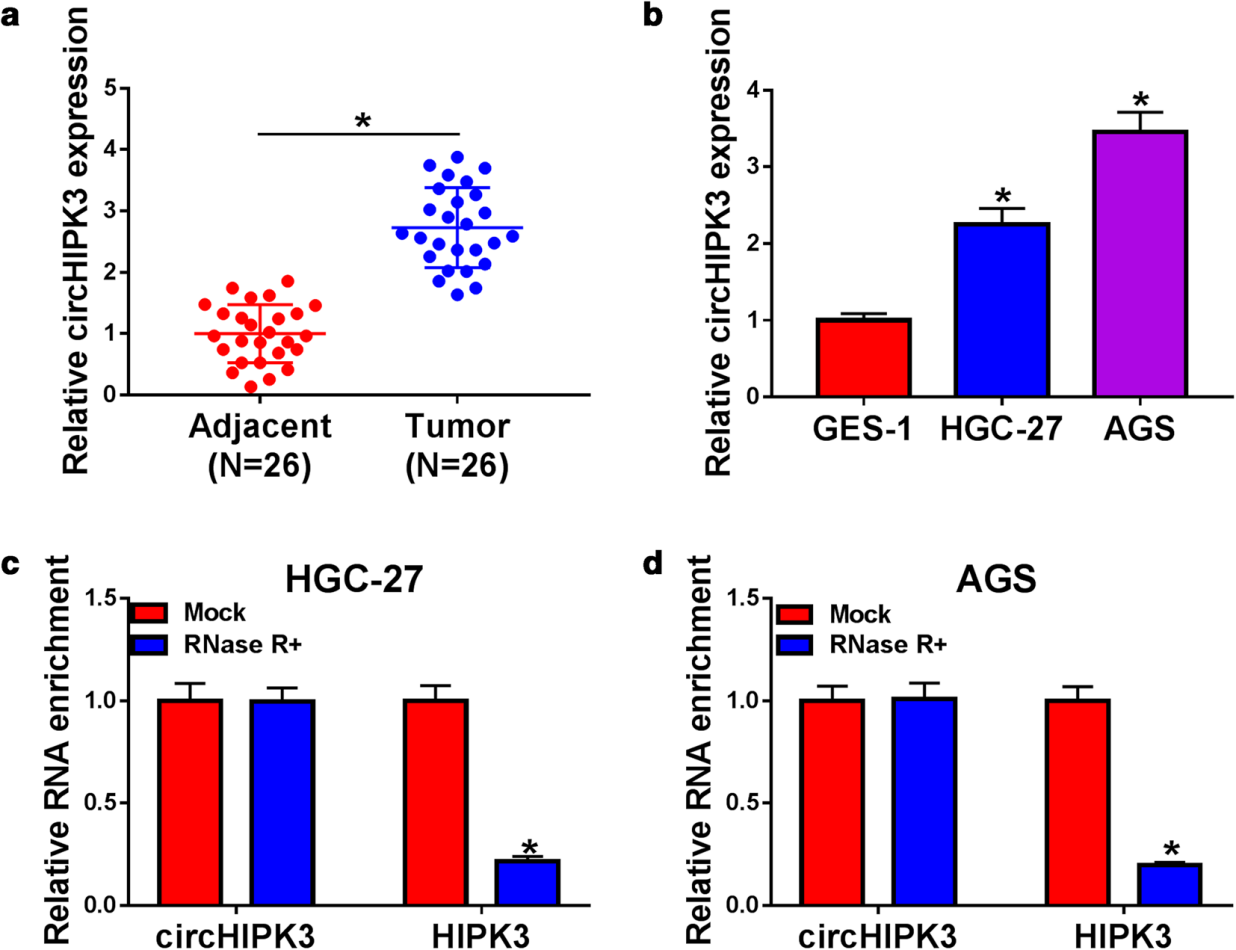
The expression level of circHIPK3 in gastric cancer tissues and cells. **a**, **b** The relative expression level of circHIPK3 was assessed with RT-qPCR assay in gastric cancer tissues and paired neighboring normal tissues, along with GES-1, HGC-27, and AGS cells. **c**, **d** RT-qPCR was applied to determine circHIPK3 and HIPK3 mRNA levels in HGC-27 and AGS cells. *P < 0.05

Initially, the results of RT-qPCR revealed that circHIPK3 was significantly upregulated in GC tissues and cells compared with neighboring normal tissues and human normal gastric epithelial cell GES-1, respectively (Fig. 1a, b). In addition, we also confirmed that circHIPK3 was more resistance to RNase R than linear HIPK3 mRNA, revealing that circHIPK3 was a circular RNA to some extent (Fig. 1c, d). Therefore, the biological role of circHIPK3 was investigated in the next experiments.

### Knockdown of circHIPK3 inhibited proliferation, migration, invasion, and glutaminolysis in GC cells

**Fig. 2:**
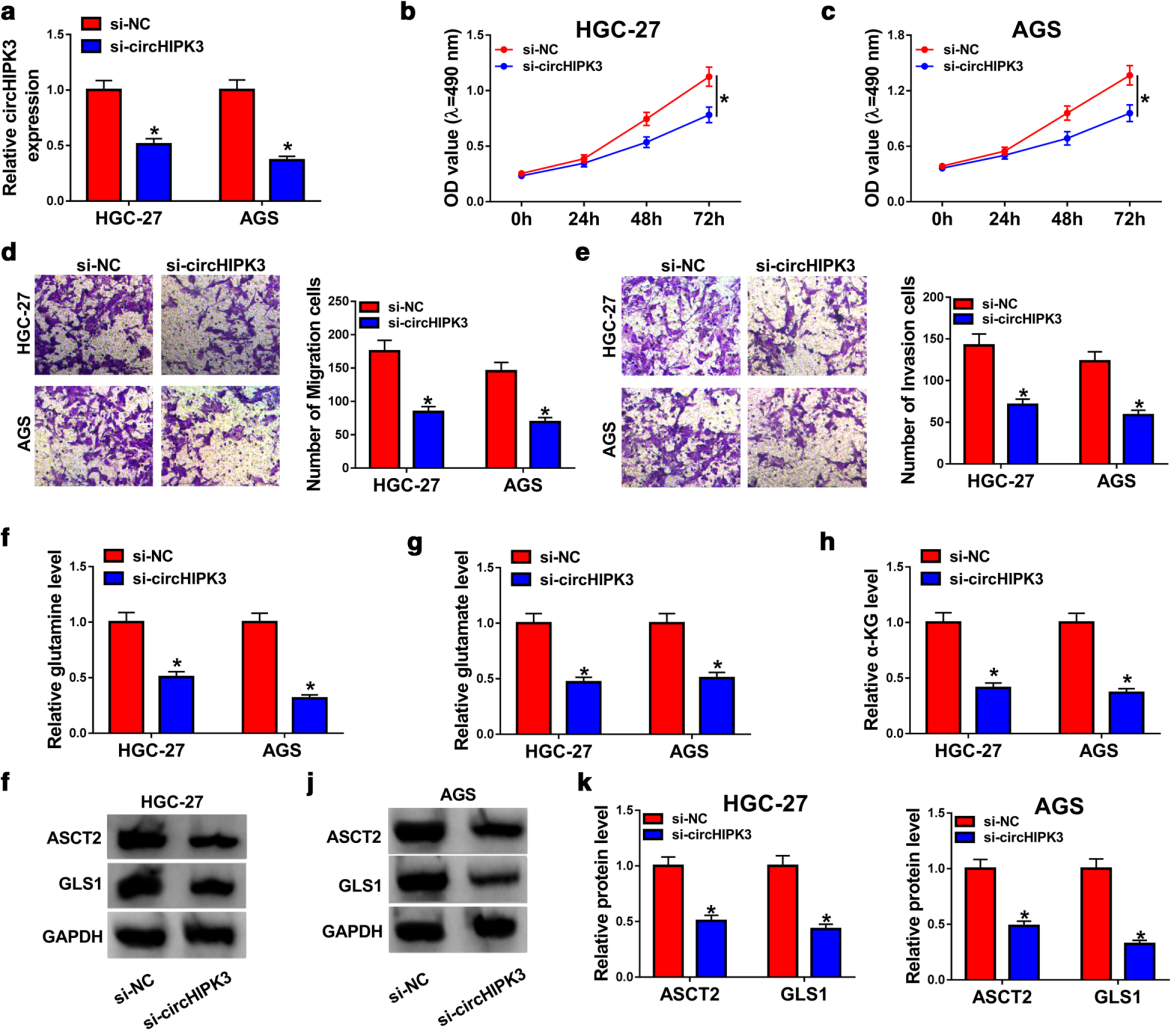
Effects of circHIPK3 silencing on proliferation, migration, invasion, and glutaminolysis in gastric cancer cells. **a**–**k** HGC-27 and AGS cells were divided into two groups: si-NC and si-circHIPK3 groups. **a** The expression level of circHIPK3 was evaluated by RT-qPCR assay in HGC-27 and AGS cells. **b**, **c** The growth curves of HGC-27 and AGS cells were analyzed using MTT assay. **d**, **e** Transwell migration and invasion assays were performed in HGC-27 and AGS cells. **f**–**h** The levels of glutamine, glutamate, and α-KG were displayed in HGC-27 and AGS cells. **i**–**k** Western blot analysis was conducted to measure ASCT2 and GLS1 levels in HGC-27 and AGS cells. *P < 0.05

As revealed in Fig. 2a, the knockdown experiments were successful using si-circHIPK3 in HGC-27 and AGS cells; the expression of circHIPK3 was decreased after transfection with si-circHIPK3. Additionally, cell viability was prominently declined at 72 h in HGC-27 and AGS cells infected with si-circHIPK3 than those cells transfected with si-NC (Fig. 2b, c). By performing transwell migration and invasion assays, we found that silencing of circHIPK3 decreased migration and invasion in HGC-27 and AGS cells (Fig. 2d, e). Furthermore, the levels of glutamine, glutamate, and α-KG were observed to assess glutaminolysis in GC cells. As presented in Fig. 2f–h, glutamine, glutamate, and α-KG were all declined in HGC-27 and AGS cells by transfection with si-circHIPK3 compared with control group. Consistently, ASCT2 and GLS1 were decreased after circHIPK3 silencing in HGC-27 and AGS cells (Fig. 2i–k). The above results revealed that silencing of circHIPK3 participated in proliferation, migration, invasion and glutaminolysis of GC cells, indicating the important role of circHIPK3 in GC.

### MiR-876-5p was a target of circHIPK3 and was decreased in GC tissues and cells

**Fig. 3:**
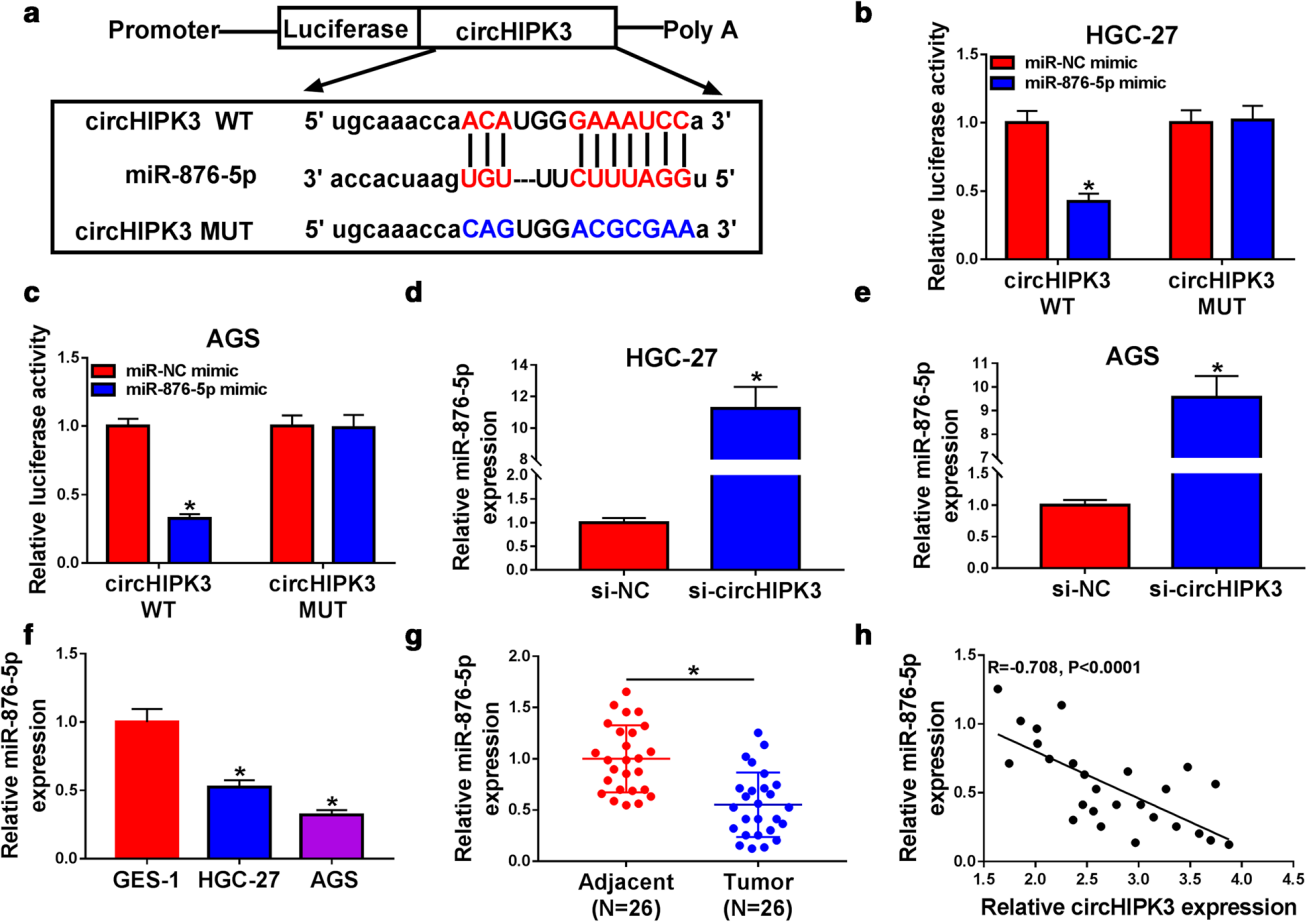
MiR-876-5p was a target of circHIPK3. **a** The binding sites of wild type circHIPK3 in miR-876-5p were shown. **b**, **c** The relative luciferase activity was assessed in HGC-27 and AGS cells by dual-luciferase report assay. **d**, **e** RT-qPCR assay was applied to analyze the expression level of miR-876-5p in HGC-27 and AGS cells transfected with si-NC or si-circHIPK3. **f**, **g** The expression level of miR-876-5p was examined by RT-qPCR assay in gastric cancer tissues and cells, along with matched control groups. **h** The correlation relationship between miR-876-5p and circHIPK3 was analyzed by Pearson’s correlation analysis in gastric cancer tissues. *P < 0.05

To identify target of circHIPK3, we searched for possible target using bioinformatics software. As presented in Fig. 3a, circHIPK3 had the complementary sequence in miR-876-5p. After that, dual-luciferase reporter vectors contained wild type or mutant circHIPK3 were constructed, and HGC-27 and AGS cells were co-transfected with indicated vectors and miR-876-5p mimic or miR-NC. The luciferase activity of circHIPK3 WT was inhibited in miR-876-5p mimic group, while the luciferase activity of circHIPK3 MUT had no difference between miR-876-5p and miR-NC group (Fig. 3b, c). In addition, knockdown of circHIPK3 enhanced miR-876-5p level in HGC-27 and AGS cells (Fig. 3d, e). Importantly, miR-876-5p showed lower expression in GC cells and tissues with respect to GES-1 cells and adjacent normal tissues, respectively (Fig. 3f, g). An adverse connection between circHIPK3 and miR-876-5p was observed in GC tissues (Fig. 3h). Therefore, circHIPK3 negatively regulated miR-876-5p expression in GC cells.

### Silencing of circHIPK3 impeded proliferation, migration, invasion and glutaminolysis of GC cells by upregulation of miR-876-5p

**Fig. 4:**
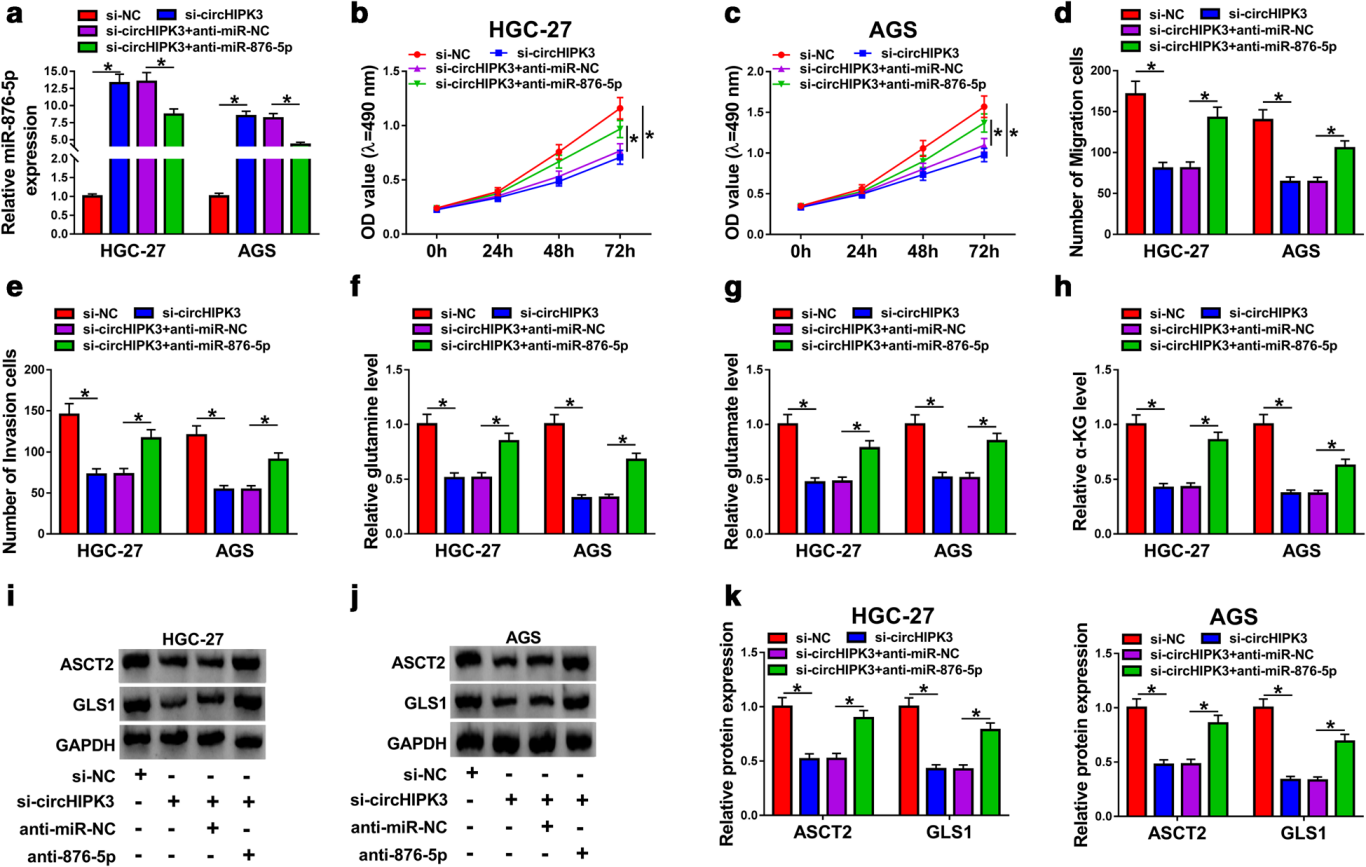
Knockdown of circHIPK3-mediated effects on proliferation, migration, invasion, and glutaminolysis were overturned by depletion of miR-876-5p in gastric cancer cells. **a**–**k** HGC-27 and AGS cells were transfected with si-NC, si-circHIPK3, si-circHIPK3 + anti-miR-NC, or si-circHIPK3 + anti-miR-876-5p. **a** The expression level of miR-876-5p was estimated by RT-qPCR assay in HGC-27 and AGS cells. **b**, **c** MTT assay was used to detect the cell viability of HGC-27 and AGS cells. **d**, **e** Transwell assay was performed to assess migration and invasion abilities of HGC-27 and AGS cells. **f**–**h** The levels of glutamine, glutamate, and α-KG were examined in HGC-27 and AGS cells. **i**–**k** The protein expression levels of ASCT2 and GLS1 in HGC-27 and AGS cells were assessed by western blot analysis. *P < 0.05

It has been confirmed that knockdown of circHIPK3 enhanced miR-876-5p level in HGC-27 and AGS cells, while it was abolished in HGC-27 and AGS cells by transfection with anti-miR-876-5p (Fig. 4a). Importantly, depletion of miR-876-5p abolished the inhibitory effect on proliferation in HGC-27 and AGS cells caused by si-circHIPK3 (Fig. 4b, c). Transwell analysis suggested that downregulation of miR-876-5p could rescue loss of migration and invasion capabilities caused by circHIPK3 knockdown (Fig. 4d, e). In addition, the levels of glutamine, glutamate, and α-KG were increased in circHIPK3-silencing GC cells after knockdown of miR-876-5p (Fig. 4f–h). Additionally, silencing of circHIPK3 induced the reduction in ASCT2 and GLS1 expression, while co-transfection of si-circHIPK3 and anti-miR-876-5p abolished these effects (Fig. 4i–k). Collectively, circHIPK3/miR-876-5p axis mediated proliferation, migration, invasion, and glutaminolysis of GC cells.

### PIK3R1 was regulated by circHIPK3/miR-876-5p axis in GC cells

**Fig. 5:**
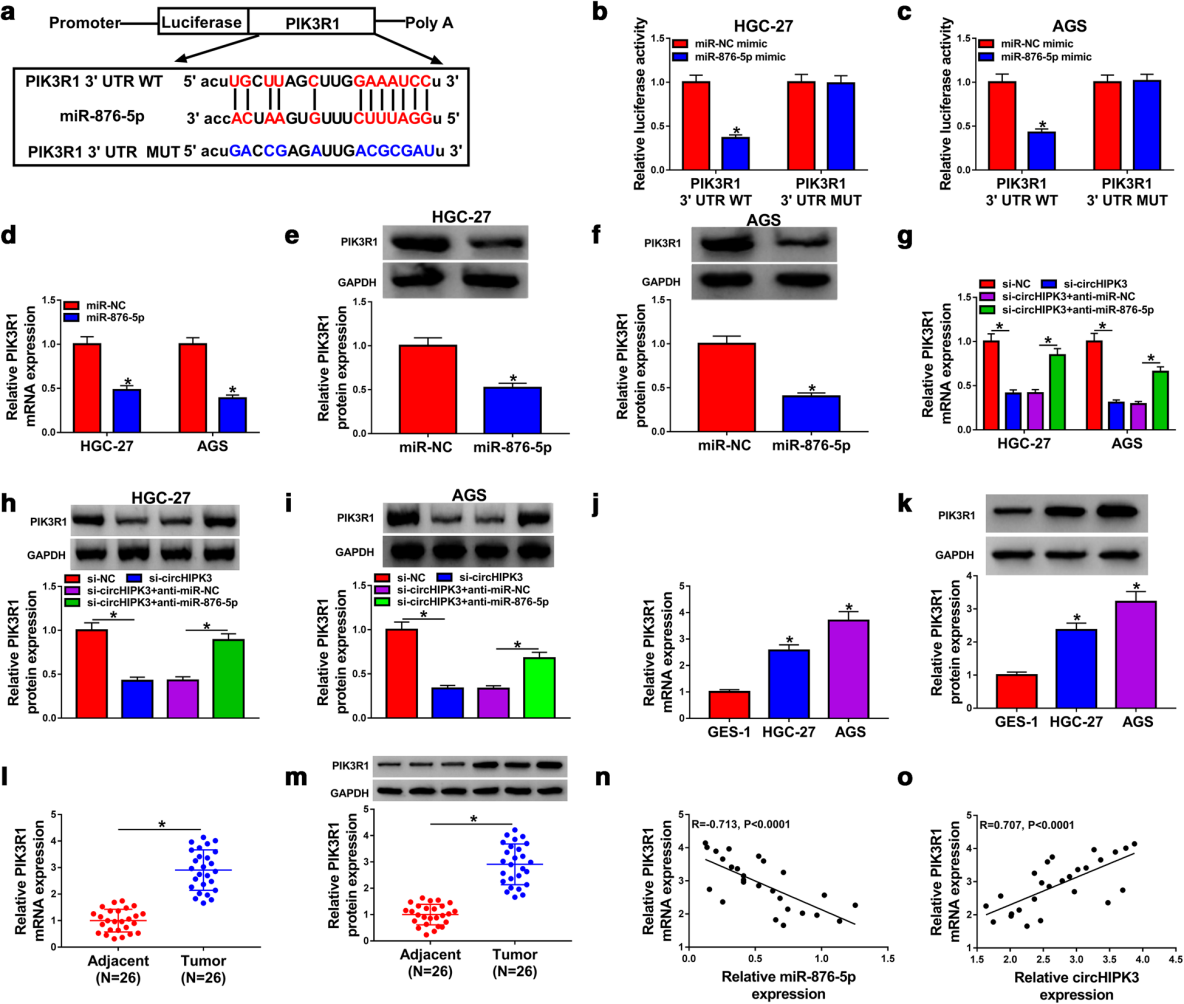
CircHIPK3 regulated PIK3R1 expression by sponging miR-876-5p. **a** Binding regions between circHIPK3 and PIK3R1, as well as matched mutation form of PIK3R1 were presented. **b**, **c** Dual-luciferase reporter assay was introduced to test the luciferase activity in HGC-27 and AGS cells. **d**–**f** RT-qPCR and western blot assays were applied to measure PIK3R1 levels in HGC-27 and AGS cells transfected with miR-NC or miR-876-5p. **g**–**i** The mRNA and protein expression levels of PIK3R1 were assessed by RT-qPCR and western blot assays in HGC-27 and AGS cells transfected with si-NC, si-circHIPK3, si-circHIPK3 + anti-miR-NC, or si-circHIPK3 + anti-miR-876-5p. **j**–**m** The expression level of PIK3R1 was examined by RT-qPCR and western blot assays in gastric cancer tissues and cells. **n**–**o** The correlation relationship between PIK3R1 and miR-876-5p or circHIPK3 was assessed by Pearson’s correlation analysis in gastric cancer tissues. *P < 0.05

As shown in Fig. 5a, we constructed luciferase reporter vectors PIK3R1 3′UTR WT contained complementary sequence to miR-876-5p, with PIK3R1 3′UTR MUT as control. Moreover, miR-876-5p mimic declined luciferase activity of PIK3R1 3′UTR WT instead of PIK3R1 3′UTR MUT in HGC-27 and AGS cells, implying that PIK3R1 was a direct target of miR-876-5p (Fig. 5b, c). The levels of PIK3R1 were decreased in HGC-27 and AGS cells after transfection with miR-876-5p, including mRNA and protein expression (Fig. 5d–f). What’s more, transfection with anti-miR-876-5p overturned the decrease in PIK3R1 expression in HGC-27 and AGS cells caused by silencing of circHIPK3 (Fig. 5g–i). We also confirmed that mRNA and protein expression levels of PIK3R1 were upregulated in GC tissues and cells than that in matched controls (Fig. 5j–m). Besides, PIK3R1 was observed to be negatively correlated with miR-876-5p, while positively correlated circHIPK3 expression in GC tissues (Fig. 5n, o). In summary, these results revealed that circHIPK3 regulated PIK3R1 by sponging miR-876-5p in GC.

### Overexpression of PIK3R1 overturned the inhibitory effects on proliferation, migration, invasion, and glutaminolysis of GC cells caused by overexpression of miR-876-5p

**Fig. 6:**
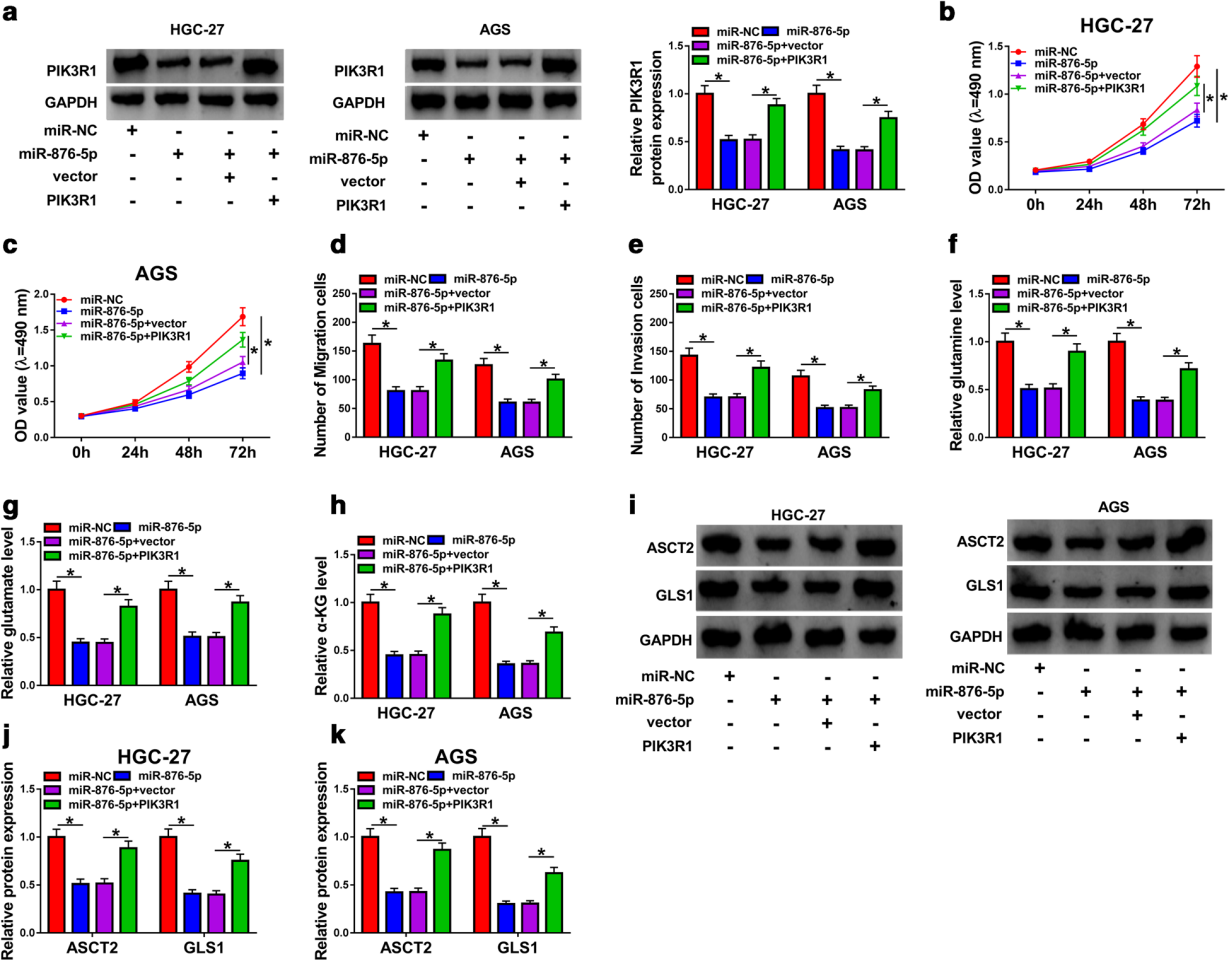
Overexpression of miR-876-5p inhibited proliferation, migration, invasion and glutaminolysis of gastric cancer cells by affecting PIK3R1. **a**–**k** HGC-27 and AGS cells were transfected with miR-NC, miR-876-5p, miR-876-5p +vector, or miR-876-5p + PIK3R1. **a** The protein expression level of PIK3R1 was measured by western blot assay in HGC-27 and AGS cells. **b**, **c** The cell proliferation was assessed by MTT assay in HGC-27 and AGS cells. **d**, **e** The cell migration and invasion were measured by transwell assay in HGC-27 and AGS cells. **f**–**h** Glutaminolysis of HGC-27 and AGS cells was determined by measuring the levels of glutamine, glutamate, and α-KG. **i**–**k** The expression levels of ASCT2 and GLS1 were examined by western blot analysis in HGC-27 and AGS cells. *P < 0.05

The results of western blot assay indicated that the upregulation of miR-876-5p inhibited PIK3R1 expression, which was reversed by transfection with PIK3R1 (Fig. 6a). The decrease of cell proliferation in miR-876-5p-overexpression cells was abrogated by upregulation of PIK3R1 (Fig. 6b, c). Moreover, transwell analysis displayed that co-transfection with miR-876-5p and PIK3R1 rescued loss of migration and invasion abilities induced by miR-876-5p (Fig. 6d, e). The overexpression of PIK3R1 could obviously increase glutamine, glutamate, and α-KG levels in HGC-27 and AGS cells with miR-876-5p overexpression (Fig. 6f–h). Interestingly, miR-876-5p-induced inhibitory effects on ASCT2 and GLS1 expression was abrogated by overexpression of PIK3R1 in both HGC-27 and AGS cells (Fig. 6i–k). Conclusively, our results revealed that miR-876-5p regulated growth, migration, invasion and glutaminolysis by targeting PIK3R1 in GC cells.

### Knockdown of circHIPK3 inhibited GC tumor growth

**Fig. 7:**
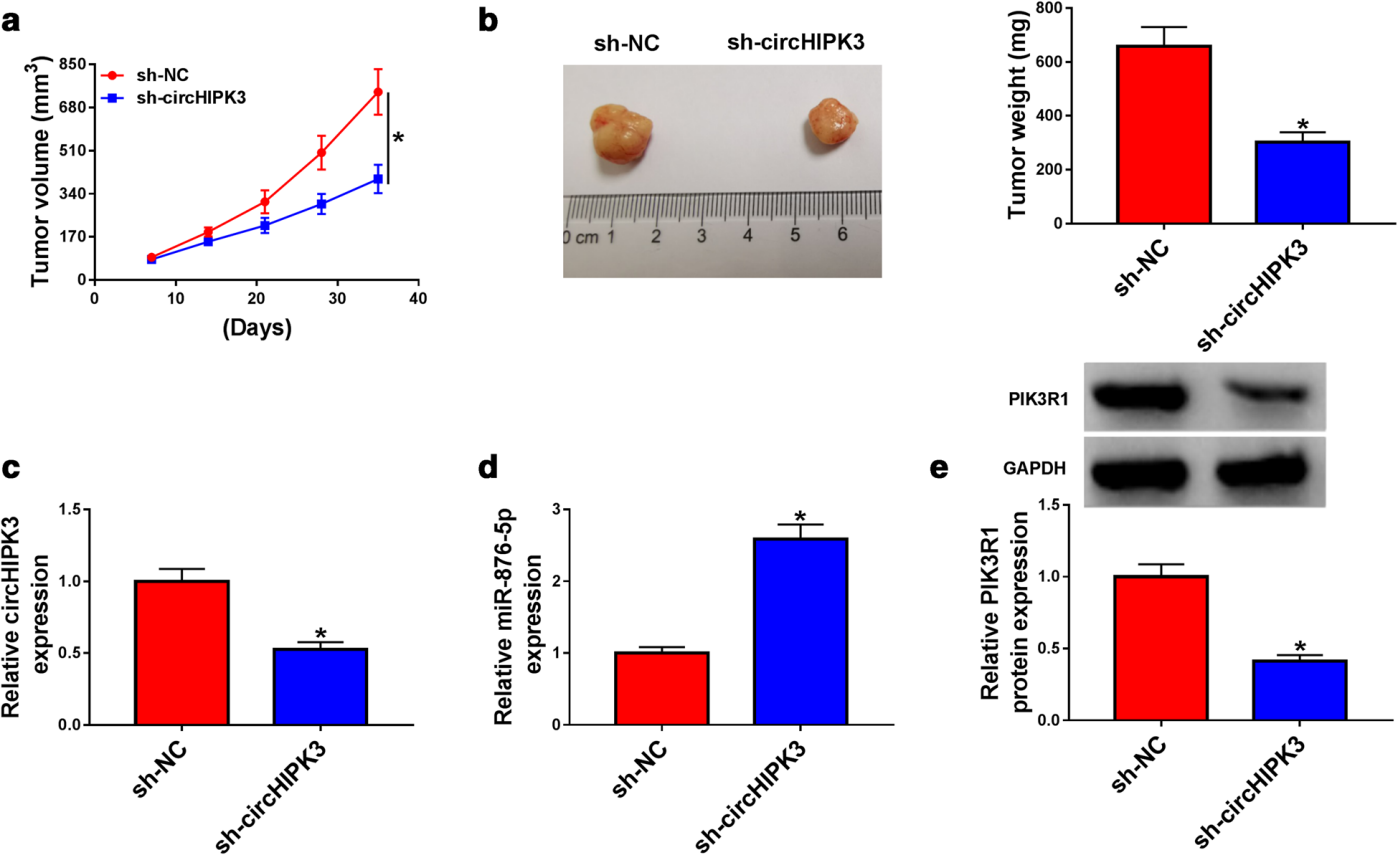
Silencing of circHIPK3 impeded tumor growth in vivo. **a**, **b** The growth curves and weight of xenograft tumors were shown. **c**, **d** The expression levels of circHIPK3 and miR-876-5p were analyzed with RT-qPCR assay in dissected tumor tissues. **e** Western blot assay was used to examine the expression level of PIK3R1 in dissected tumor tissues. *P < 0.05

To probe the effect of circHIPK3 on GC cells proliferation in vivo, a xenograft tumor model in nude mice was established. As displayed in Fig. 7a, b, downregulation of circHIPK3 led to the smaller tumor volumes and weight than volumes tissues from control group. Furthermore, circHIPK3 was decreased, while miR-876-5p was increased in xenografts from sh-circHIPK3 group compared with sh-NC group (Fig. 7c, d). The western blot assay results indicated that suppression of circHIPK3 impeded PIK3R1 expression in xenografts (Fig. 7e). Synthetically, silencing of circHIPK3 suppressed GC growth in vivo.

## Discussion

Conclusively, our study suggested the cancerogenic role of circHIPK3 in GC, and the silencing of circHIPK3 impeded GC cells proliferation, migration, invasion, as well as inhibited the tumor growth in vivo. Importantly, we found that a novel regulatory mechanism of circHIPK3/miR-876-5p/PIK3R1 axis was involved in GC development. Furthermore, evidence has shown that survive and proliferation of cancer cells were highly dependent on glutaminolysis. ASCT2, high-affinity glutamine importer, was involved in amino acid transport and metabolism in cancer [16]. Glutamine was metabolized by rate-limiting enzyme GLS1, and eventually converted to α-KG [17]. Therefore, the levels of ASCT2, GLS1, and α-KG were closely correlated with glutaminolysis. In this paper, knockdown of circHIPK3 inhibited ASCT2, GLS1, and α-KG levels, suggesting silencing of circHIPK3 inhibited glutaminolysis in GC cells.

Recently, multiple studies have revealed an extensive landscape of circRNAs with exquisite regulation of the development of malignant tumors through interaction with miRNAs [18, 19]. The studies had shown that circHIPK3 functioned as an oncogene in malignant tumors [6, 7, 20]. By the bioinformatics prediction, we noticed that circHIPK3 had the binding sites on miR-876-5p. Subsequently, the direct association between circHIPK3 and miR-876-5p was verified using dual-luciferase reporter assay. In addition, circHIPK3 was adversely connected with miR-876-5p expression in GC tissues. As we expected, knockdown of miR-876-5p could counteract the inhibitory effects on growth, mobility, and glutaminolysis in GC cells caused by silencing of circHIPK3. Analogously, Sang et al. revealed that ciRS-7 stimulated esophageal squamous cell carcinoma progression by sponging miR-876-5p [21]. In addition, previous study by Xie et al. has unveiled the anti-tumor role of miR-876-5p in osteosarcoma by targeting c-Met [22].

For investigating the downstream target of the circHIPK3-miR-876-5p axis, dual-luciferase reporter analysis was used and identified that PIK3R1 was a functional target of miR-876-5p. The PI3K family, belong to kinases of inositol and phosphatidylinositol, were oncogenes according pervious report [23]. The regulatory subunit (p85a), the composition of PI3K, was encoded by PIK3R1 [24], while PI3K signaling was involved in multiple human diseases, including, inflammation, malignant tumor, and diabetes [25, 26]. We supposed that PIK3R1 was an oncogene in malignant tumors. Surely, decrease of PIK3R1 suppressed proliferation and invasion in glioblastoma multiform cells [27]. Moreover, reduction of PIK3R1 induced apoptosis and cycle arrest in colorectal cancer cells by repressed cyclin D1 expression [28]. Analogously, Huang et al. confirmed that miR-486-5p played a tumor-inhibitory role by suppressing PI3K/AKT pathway activation via targeting PIK3R1 [29]. However, the potential mechanism of PI3K/AKT regulation in GC by PIK3R1 should be further analyzed According above researches, PIK3R1 might be a disadvantageous index for prediction of GC. Not surprisingly, Xia et al. discovered PIK3R1 level was increased by nuclear paraspeckle assembly transcript 1 in GC and afterward stimulated GC progression [30]. In this study, PIK3R1 was increased in GC and adversely correlated with that of miR-876-5p expression in GC tissues. Furthermore, functional cellular experiments indicated that the upregulation of PIK3R1 increased the proliferation, migration, invasion, and glutaminolysis in miR-876-5p-overexpression GC cells.

In the current study, circHIPK3 was upregulated in GC tissues and cells after RT-qPCR examination. The following loss-of-function assays revealed that silencing of circHIPK3 impaired proliferation, migration, invasion and glutaminolysis capacities of GC cells. Mechanistically, circHIPK3 exerted its cancerogenic functions through miR-876-5p/PIK3R1 axis in GC.

## Conclusion

In summary, circHIPK3 was upregulated in GC samples and cells when compared with adjacent noncancerous tissues and gastric epithelial cells, correspondingly. Functional experiments indicated that silencing of circHIPK3 impeded GC progression by inhibiting proliferation, migration, invasion and glutaminolysis of GC cells through miR-876-5p/PIK3R1 axis, suggesting a potential therapeutic strategy for GC in the future.

## Data Availability

All data generated or analysed during this study are included in this published article.
